# Supplementary material for: Polymorphisms in Pfkelch13 domains before and after the introduction of artemisinin-based combination therapy in Southwest Nigeria
Source: PLoS One. 2025 Mar 31;20(3):e0316479. doi: 10.1371/journal.pone.0316479 (PMC11957316; doi:10.1371/journal.pone.0316479)

Samples: 13173
Bases: 1119
Average spacing: 12

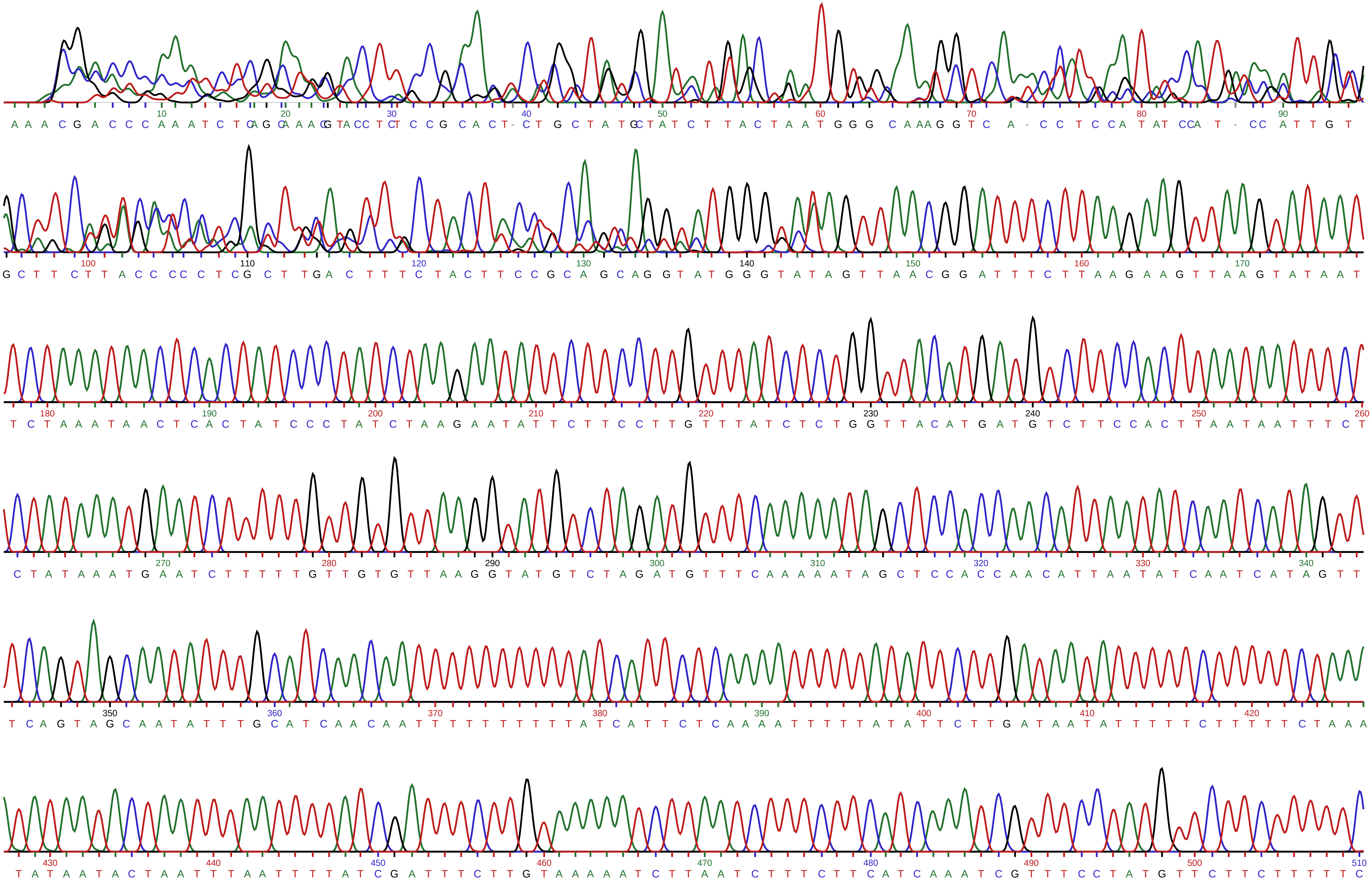

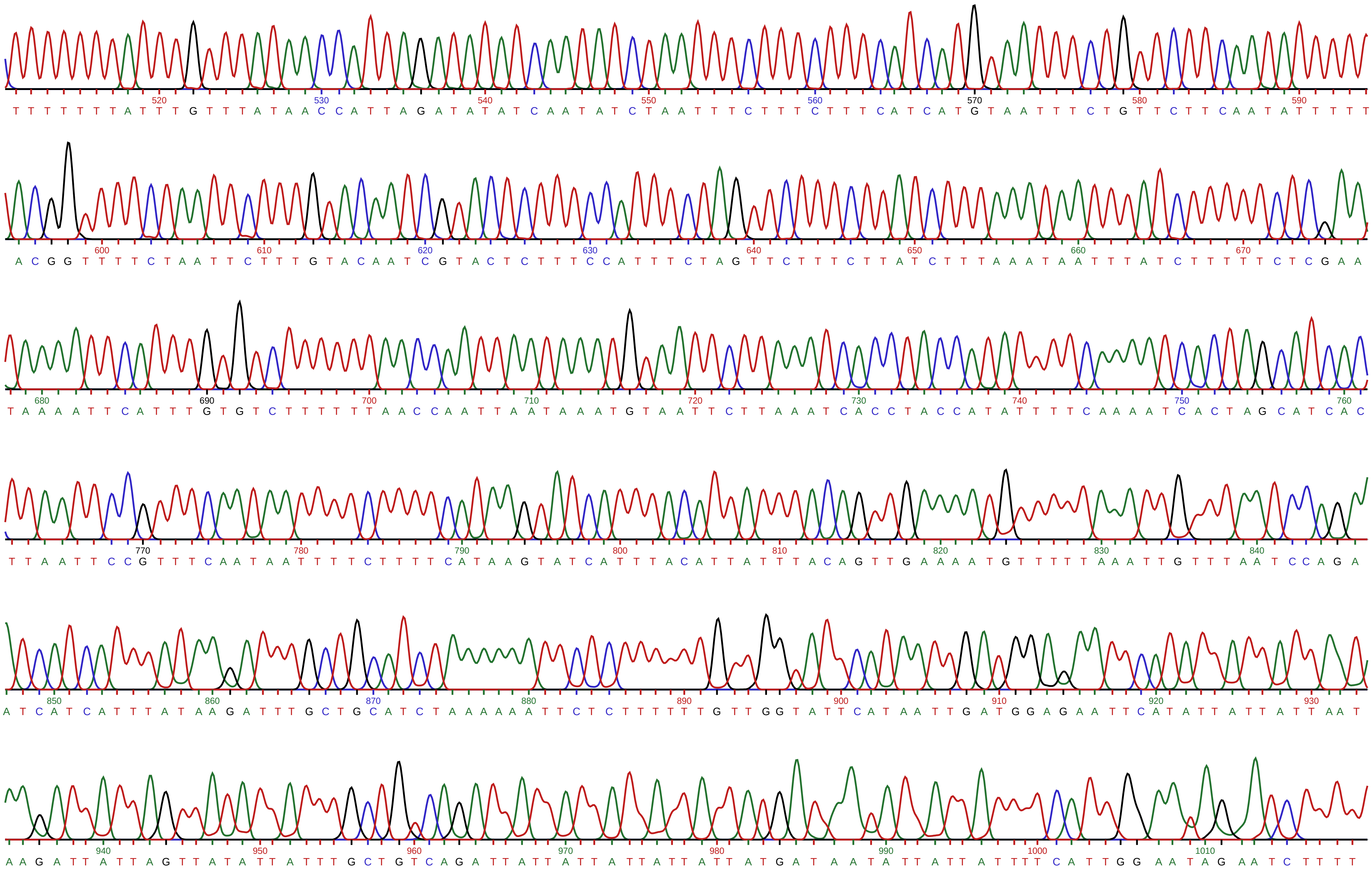
520 530 540 550 560 570 580 590
T T T T T T T A T T T G T T T A T A A C C A T T A G A T A T A T C A A T A T C T A A T T T C T T T C T T T C A T C A T G T A A T T T C T G T T C T T C A A T A T T T T T
600 610 620 630 640 650 660 670
A C G G T T T T C T A A T T C T T T G T A C A A T C G T A C T C T T T C C A T T T C T A G T T C T T T C T T A T C T T T A A A T A A T T T A T C T T T T T C T C G A A
680 690 700 710 720 730 740 750 760
T A A A A T T C A T T T G T G T C T T T T T T A A C C A A T T A A T A A A T G T A A T T C T T T A A A T C A C C T A C C A T A T T T T C A A A A T C A C T A G C A T C A C
770 780 790 800 810 820 830 840
T T A A T T C C G T T T C A A T A A T T T T C T T T T C A T A A G T A T C A T T T A C A T T A T T T A C A G T T G A A A A T G T T T T T A A A T T G T T T A A T C C A G A
850 860 870 880 890 900 910 920 930
A T C A T C A T T T A T A A G A T T T G C T G C A T C T A A A A A A T T C T C T T T T T T G T T G G T A T T C A T A A T T G A T G G A G A A T T C A T A T T A T T A T T A A T
940 950 960 970 980 990 1000 1010
A A G A T T A T T A G T T A T A T T A T T T G C T G T C A G A T T A T T A T T A T T A T T A T T A T T A T G A T A A T A T T A T T A T T T T C A T T G G A A T A G A A T C T T T T

Samples: 13173
Bases: 1119
Average spacing: 12

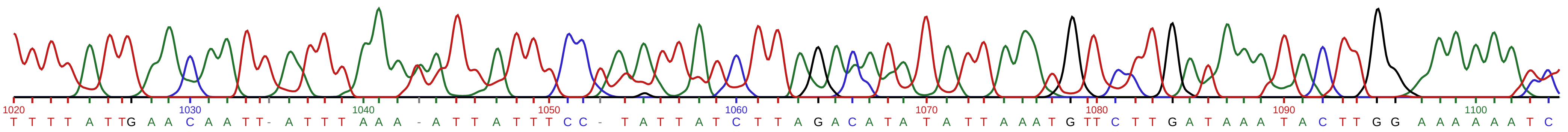

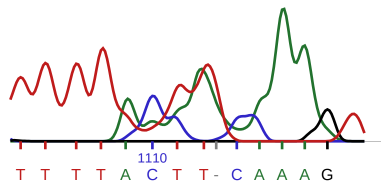

Supplement: Supporting information 2 — (ZIP) [file pone.0316479.s002.zip › 064KN2R_PREMIX_Plate_KELCH2_G04.pdf]
